# Investigating Neural Efficiency in the Visuo-Spatial Domain: An fmri Study

**DOI:** 10.1371/journal.pone.0051316

**Published:** 2012-12-12

**Authors:** Ilona Lipp, Mathias Benedek, Andreas Fink, Karl Koschutnig, Gernot Reishofer, Sabine Bergner, Anja Ischebeck, Franz Ebner, Aljoscha Neubauer

**Affiliations:** 1 Institute of Psychology, University of Graz, Graz, Austria; 2 Cardiff University Brain Research Imaging Centre (CUBRIC), School of Psychology, Cardiff University, Cardiff, United Kingdom; 3 Department of Radiology, Medical University of Graz, Graz, Austria; University Of Cambridge, United Kingdom

## Abstract

The neural efficiency hypothesis postulates an inverse relationship between intelligence and brain activation. Previous research suggests that gender and task modality represent two important moderators of the neural efficiency phenomenon. Since most of the existing studies on neural efficiency have used ERD in the EEG as a measure of brain activation, the central aim of this study was a more detailed analysis of this phenomenon by means of functional MRI. A sample of 20 males and 20 females, who had been screened for their visuo-spatial intelligence, was confronted with a mental rotation task employing an event-related approach. Results suggest that less intelligent individuals show a stronger deactivation of parts of the default mode network, as compared to more intelligent people. Furthermore, we found evidence of an interaction between task difficulty, intelligence and gender, indicating that more intelligent females show an increase in brain activation with an increase in task difficulty. These findings may contribute to a better understanding of the neural efficiency hypothesis, and possibly also of gender differences in the visuo-spatial domain.

## Introduction

The biological background of intelligence has fascinated cognitive science for decades. Researchers have been looking for brain areas that are relevant for higher cognitive functioning and that are responsible for differences between individuals who are better in solving cognitive tasks as compared to those performing worse. Most studies investigating that issue correlate intelligence measures with different anatomical and functional characteristics of the brain, including gray and white matter volume, white matter density, brain metabolites etc., and all of them seem to play a role (for a review see [1]).

One theory on the “localization” of intelligence in the brain has been posited by Jung and Haier [2]. Based on a review of 37 neuroimaging studies, they suggest a number of gray matter areas and their white matter connections that may play a crucial role in intelligence. In their so-called parieto-frontal integration theory (P-FIT) they postulate subsequent steps of reasoning, in which different brain areas are involved: after the processing of sensory information by temporal and occipital lobes (specifically BAs 18, 19, 37, 22), information is assumed to be transmitted to the parietal cortex (BAs 40, 7, 39) and extracted there. Regions of the frontal cortex (BAs 6, 9, 10, 45–47) are believed as being responsible for the achievement of a solution of a given cognitive task, while response selection could take place in the anterior cingulate (BA 32). The P-FIT model specifically focuses on brain regions related to general intelligence. The observed activation during an intelligence task is expected to depend also on distinct task requirements. Mental rotation tasks, which are commonly used to study spatial intelligence, have been reported to consistently activate superior parietal, frontal, and the inferior temporal regions [3].

Another important concept in this field, known as the “neural efficiency hypothesis of intelligence”, is based on the observation that under certain experimental conditions less intelligent participants activate their brains to a greater extent, therefore showing less efficiency when solving a task [4]. The idea of neural efficiency was first introduced by Haier et al. [5], who observed a negative relationship between cognitive functioning and activation over the whole brain as measured by positron emission tomography (PET). In a subsequent study, Haier et al. [6] were able to demonstrate that glucose metabolism can be decreased by practice, and that this effect is observable in different brain areas. These empirical findings led to the specification of the neural efficiency hypothesis, postulating that more intelligent people are more likely to focus their activation on task-relevant areas, whereas less intelligent people activate their brains in a more distributed way [6].

Meanwhile, the neural efficiency hypothesis has been tested in many studies employing different neurophysiological methods and a broad variety of different cognitive task demands. Most studies have used electroencephalography (EEG) for measuring brain activation, and one commonly used approach in this context is the method of event-related-(de)synchronisation of EEG brain activity (ERS/ERD; [7]). This method focuses on synchronization or desynchronization of EEG activity, particularly in the upper alpha-band (10–12 Hz). The neural efficiency hypothesis has been supported by numerous EEG studies (e.g. [8]). However, there is also contradictory evidence, which might be explained by some factors that can complicate the activation-intelligence relationship, such as task difficulty, state of learning, brain area, gender and task modality [4]. Furthermore, two EEG studies reported an interaction between gender and task-modality.

In the study of Neubauer et al. [9], three different variants of Posner's letter matching task [10] were used, the original verbal task, a spatial version using congruently or incongruently rotated arrows and a numerical task version using Arabic numbers and dice faces. Results indicated that neural efficiency varied as a function of gender and task modality. More intelligent females showed less cortical activity than less intelligent females (i.e., neural efficiency) during the verbal version of the Posner paradigm, while no such differences were apparent in the visuo-spatial and numerical task variants. On the other hand, brighter males showed less cortical activity than less intelligent males only while working on the visuo-spatial version of the paradigm.

In 2005, Neubauer et al. [11] were able to replicate this finding in a subsequent study, which additionally assessed the level of intelligence in the respective domains (i.e., verbal, visuo-spatial, and numerical) rather than general intelligence only. For males, the expected negative correlation between intelligence and activation was only found during the visuo-spatial task and only when visuo-spatial intelligence was considered. In contrast, neural efficiency in females was apparent during the verbal task, but only when verbal intelligence was considered. Also, the brain areas in which neural efficiency was found differed between both genders. In females, intelligence and activation were inversely correlated at centroparietal and parietooccipital electrode positions, while in males neural efficiency was found in frontal regions. This study aims to further investigate this gender-modality interaction with specific regard to brain regions.

One tentative interpretation for the gender-task modality interaction could be that the visuo-spatial task is more difficult for women, while the verbal task is harder for men, and that neural efficiency is only found for easy to moderate task difficulty [4], [12]. Mental rotation tasks are commonly known as producing the largest gender differences in performance favoring males [13]–[15]. However, since gender differences in brain activation during visuo-spatial tasks have been frequently reported [16]–[20] it might also be considered that females and males use different brain areas when solving such tasks. Further evidence for gender differences with regard to the intelligence-activation relationship was provided by Haier et al. [21] who found a positive relationship between temporal activation and aptitude during a math task, but for males only. There also seem to be gender differences in the relationship between anatomical features of the brain and intelligence, as suggested by Haier et al. [22] who found that in females white matter is higher correlated and grey matter less correlated to intelligence than in males.

So far, functional magnetic resonance imaging (fMRI) studies that report correlations between brain activation and intelligence show mixed results. It has to be mentioned that most of these studies were not specifically designed to investigate neural efficiency. Some studies found evidence for a negative relationship between brain activation and intelligence, and most of the correlations were found in frontal and parietal regions [23]–[29]. Some fMRI studies report a positive rather than a negative relationship between brain activation and cognitive abilities. Again, correlations were found in frontal as well as parietal brain regions during different kinds of tasks [30]–[33]. Other studies showed positive as well as negative correlations in different areas, also suggesting that more intelligent people might differ from less intelligent people in their activation patterns [2], [34]–[39]. Taken together, most of the reported correlations between brain activation and measures of intelligence were found in frontal and parietal areas, the areas that have been identified as being most relevant for individual differences in intelligence according to the parieto-frontal integration theory of intelligence [39].

The specific aim of this study is to investigate the neural efficiency hypothesis (i.e., the assumed inverse relationship between brain activation and intelligence) by means of functional MRI and with special regard to gender differences. As briefly outlined above, EEG studies revealed some evidence that neural efficiency during cognitive task performance is moderated by gender and task content. In this study, we measured brain activation using fMRI in female and male adolescents during the performance of a visual-spatial task, similar to that used in previous EEG studies. In investigating brain activity related to intelligence during visuo-spatial information processing by means of fMRI, we would be able 1) to provide a further test of the neural efficiency hypothesis in using a different method of neurophysiological measurement, 2) examine the possible moderating role of gender and task difficulty, and 3) obtain a more fine-grained picture of the specific brain areas related to individual differences in intelligence.

## Methods

### Participants

Out of a larger pool of 900 adolescents, a sample of 20 males (age: *M* = 16.8, *SD* = 0.8) and 20 females (age: *M* = 16.9, *SD* = 0.6) was selected. The selection of participants was based on two criteria: First, participants should display a large variability in intellectual ability; and second, females and males should not differ significantly with respect to visuo-spatial and general intelligence test scores. The mean visuo-spatial intelligence quotient (IQ) for females was *M* = 103.48 and *M* = 105.58 for the male participants, with a standard deviation of *SD* = 15.9 and *SD* = 14.0, respectively (general intelligence scores for females were *M* = 99.08, *SD* = 14.4, for males *M* = 101.89, *SD* = 16.7). All participants were healthy, right-handed, had normal or corrected-to-normal vision, gave written informed consent (in case of underage participants parents gave written informed consent), and were paid for their participation. The study was approved by the local ethics committee of the Medical University of Graz, Austria.

### Psychometric testing

Intelligence was assessed by means of a well-established German intelligence test (Intelligenz-Struktur-Test [intelligence-structure-test] I-S-T 2000-R; [40]), which includes three verbal (finding similarities, analogies, sentence completion), three numerical (arithmetic problems, numerical series, arithmetic operations) and three visuo-spatial (assembling figures, rotation cubes, matrices) reasoning tests. This test allows the measurement of specific intellectual abilities, as well as to combine subtest-scores to one total reasoning score.

Individual subscale and reasoning scores were standardized by means of age-specific norms (15–16 year, or 17–18 year old students). Six out of the 40 fMRI participants did not complete the I-S-T 2000-R. For the analyses, their intelligence scores from the screening test of intelligence (Intelligenz-Struktur-Analyse [Intelligence Structure Analysis], Fay et al., [41]), a test that is also standardized with norm values, were used.

In addition to the intelligence measure, personality was assessed with the Neuroticism Extraversion Openness Five Factor Inventory [42] and state-anxiety was measured with the State-Trait Anxiety Inventory [43].

### Experimental task

**Figure 1:**
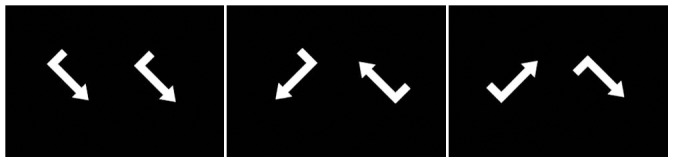
Experimental task. Left: identical condition; middle: experimental condition with two congruent arrows rotated in a 90° angle; right: experimental condition with two incongruent arrows rotated in a 90° angle.

The experimental task was a visuo-spatial variant of the standard Posner task [11] as used in the Neubauer et al. [12]. Two white arrows in different rotation angles were presented simultaneously on a black screen (Figure 1), and participants were required to judge whether, after rotation, the angled arrows were congruent or incongruent (mirror images). To solve this task, the arrows needed to be mentally rotated until they pointed in the same direction. Arrows in each pair differed by either 45°, 90°, 135° or 180°. A control condition was implemented by using identical arrows that were not rotated (0°).

### Scanning procedure

An event-related approach was used for item presentation and analysis. Forty-eight experimental and 48 control items were presented in randomized order, with each item presented only once. Half of the experimental items depicted congruent, the other half incongruent pairs of arrows. Each item was presented for 3 seconds to ensure that participants had enough time to complete the item and to obtain a high solution rate. Inter-trial intervals consisted of a fixation cross, with a variable duration between 4 and 10 seconds.

A 3.0 T Tim Trio Scanner (Siemens Medical Systems, Erlangen, Germany) with a 32 channel head coil was used. Functional images were obtained in 34 slices, in descending order. T2*-weighted functional images were obtained with a single shot gradient echo planar imaging (EPI) sequence sensitive to blood oxygen level-dependent (BOLD) contrast (repetition time [TR] 2000 ms, echo time [TE] 25 ms, voxel size 3×3 mm, matrix size 64×64, field of view: 192, flip angle 90°).

Responses were obtained by means of a response box (Current Designs, Inc, Philadelphia, USA), located in the participant's right hand. The software Presentation (Neurobehavioral Systems, Albany, CA) was used for item presentation and registration of behavioral performance (solution rate and reaction time).

### Procedure

The scanning procedure was thoroughly explained to the participants prior to the fMRI experiment. The experimental task was demonstrated on a computer outside the scanner room, and participants completed a test run consisting of ten items. The whole fMRI testing session including instructions, T_1_- and T_2_-weighted structural scans, the mental rotation task, and another cognitive task (which was used in another research context), and lasted about 80 minutes.

Participants were tested with the psychometric intelligence test on a different day. Psychometric testing was conducted as group sessions in a lecture hall at the University Campus, taking about 90 minutes.

### Analysis

Functional MRI scans were analyzed using the Software SPM8 (Wellcome Department of Imaging Neuroscience, London, UK). For each person approximately 500 functional images were obtained. Preprocessing was performed, including motion correction, slice acquisition time correction, normalization to MNI space and smoothing. A general linear model (GLM) was estimated for each participant, using the conditions ‘ROTATION’ (i.e., experimental trials requiring mental rotation), ‘IDENTICAL’ (i.e., control trials requiring no mental rotation) and ‘FIXATION’ (i.e., inter-trial fixation period) as predictors of interest. All trials were entered into the analysis, with an additional regressor coding the correctness of a participant's answer. Finally, motion parameters were included in the model to account for uncorrected motion effects.

For the analysis of task-related activation, a factorial as well as a parametric design was implemented, which is a commonly used approach [44]. In the factorial approach, task-related activation was defined as the activation found using the contrast ROTATION > IDENTICAL. Second level analysis was done by calculating a one-sample *t*-test, employing a conservative significance level of *p*<10^−8^ (corrected for family wise error [FWE]). Potential gender differences in activation patterns were analyzed with a two-sample *t*-test using gender as the between subjects factor. Results are reported for *p*<.05 (FWE corrected).

Results are only presented if they are significant on cluster level (*p*<.05, FWE corrected) and exceed a minimum cluster size of 100 for task-relevant activation, and 50 for gender differences and intelligence influences. The significant clusters of the contrast ROTATION > IDENTICAL were used for the construction of functionally defined ROIs of task-related activation. Percent signal change values (for the ROTATION condition) within the ROIs were used for further analyses. For correlations between intelligence and the percent signal change values the common significance level of *p*<.05 was used.

For the parametric approach, task difficulty (45°, 90°, 135°, 180°) was included in the GLM as a covariate of interest, allowing to identify areas where activation covaried with rotation angle. Results are reported for *p*<.05 (FWE corrected). The interaction between intelligence and task difficulty was investigated by adding intelligence as a covariate in the second level analysis. Results are reported for *p*<.001 (uncorrected), and presented when they are significant on cluster level (*p*<.05, FWE corrected) and exceed a minimum cluster size of 50. The less strict significance level for the relationship with intelligence was chosen because of limited power in the analysis of higher-order effects.

## Results

### Behavioral results

Visuo-spatial intelligence was significantly correlated with performance in the mental rotation task. More intelligent individuals had a higher solution rate (*r*[38] = .46) and shorter reaction times (*r*[38] = −.32). There were no gender differences in task performance, neither in solution rate (*M*_fem_ = .86, *M*_male_ = .86, *t*[38] = 0.07, *ns)* nor in reaction times (*M*_fem_ = 1.65 s; *M*_male_ = 1.57 s, *t*[38] = 0.91, *ns)*.

Reaction time increased significantly with an increase of rotation angle (*F*[2.0, 79.3] = 74.3, *p*<.001, *n* = 40, η^2^_partial_ = .66, Greenhouse-Geisser corrected). Mean reaction times (with standard deviations in parentheses) for rotation angles 45°, 90°, 135° and 180° were 1.32 s (0.28), 1.52 s (0.28), 1.76 s (0.35) and 1.82 s (0.38), respectively. Solution rate decreased significantly with an increase of rotation angle (*F*[2.1, 80.0] = 37.8, *p*<.001, *n* = 40, η^2^_partial_ = .49). Mean solution rates (with standard deviations in parentheses) for rotation angles 45°, 90°, 135° and 180° were .97 (.06), .93 (.09), .84 (.13) and .74 (.19), respectively. The increase in reaction time and decrease in solution rate indicate that rotation angle reflects task difficulty.

### Task-related activation as revealed by the contrast ROTATION > IDENTICAL

**Figure 2:**
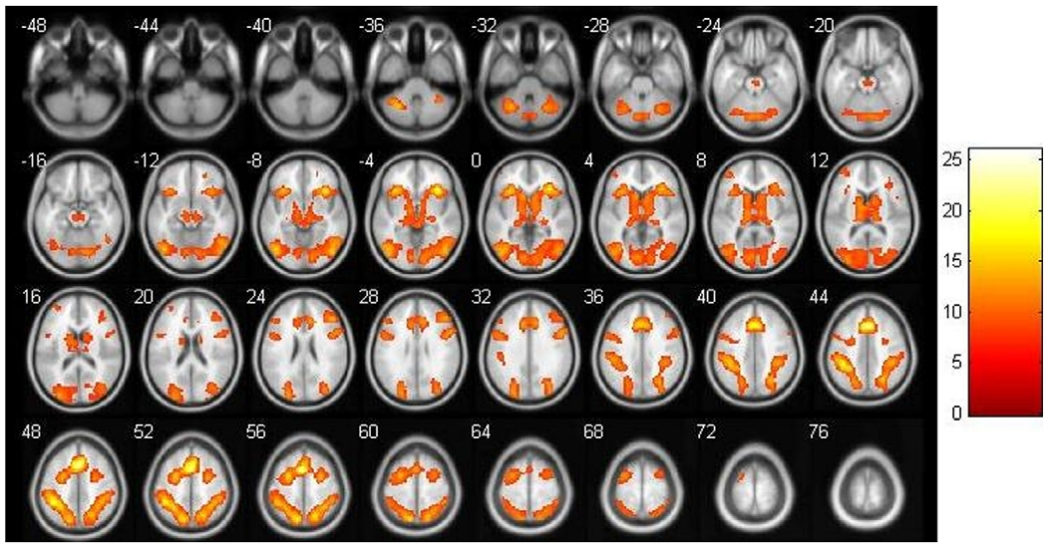
Task-related activation. Activation is shown for the contrast experimental > ident, with a significance level of P<10^−8^ (FDR).

The contrast ROTATION > IDENTICAL revealed eight clusters of significant task-related activation (see Table 1, Figure 2). There was no significant gender difference in task-related activation, only a tendency for stronger activation in the right lingual gyrus for males [*p* = .077 (FWE corrected); x, y, z: 15, −55, −5; *T*_peak_ = 4.35, k = 67].

**Table 1:** Task-related activation. Presented are significant clusters identified in the contrast ROTATION > IDENTICAL (*p*<10^−8^, FWE corrected, k>100).

Coordinates			*P*_Cluster_ (FWE)	*T*_Peak_	Cluster size (*k*)	Location
x	Y	z				
3	20	46	.000	25.89	626	Supplementary motor area (SMA)
−39	−40	46	.000	20.53	926	Left inferior parietal
33	23	−5	.000	20.13	179	Right inferior frontal
18	−67	55	.000	17.00	552	Right superior parietal
−33	20	−2	.000	16.07	125	Left insula
45	−76	−8	.000	14.69	151	Right inferior occipital
−9	−16	10	.000	13.17	144	Thalamus
21	−82	−2	.000	12.66	115	Right lingual gyrus

The reverse contrast IDENTICAL > ROTATION revealed areas that showed stronger activation during the non-rotation condition than during the rotation condition. At the significance level of 10^−4^ (*k*>100) a significant difference in brain activation was observed in a region of the posterior cingulate/precuneus [x, y, z: 9, −49, 34; *p* (FWE) = .000, *T*_peak_ = 10.20, k = 157]. Percent signal change was also extracted from this cluster. The mean percent signal change value was negative, indicating a deactivation of this brain region.

#### Correlations with intelligence

**Figure 3:**
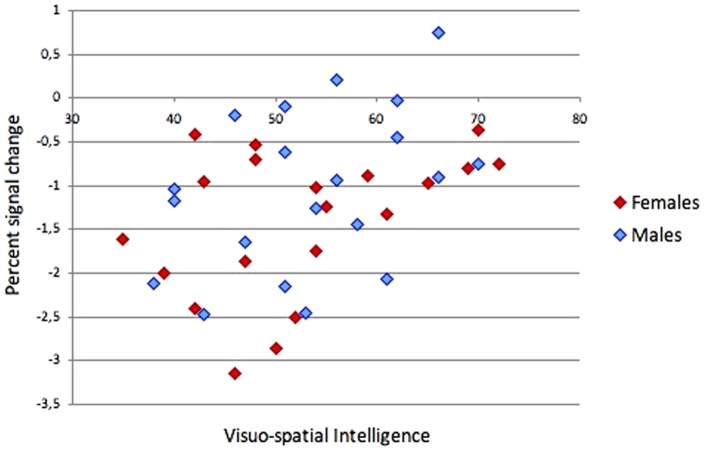
Relationship between intelligence and brain activation. Scatter plot of the relationship between intelligence and %SC values in posterior cingulate and precuneus for both genders.

Correlations were computed between visuo-spatial intelligence and percent signal change in each of the nine task-related ROIs. No significant correlations were found in regions showing task-related increases of brain activation in the total sample, or in separate analyses for females and males. However, signal change values in the region within the posterior cingulate/precuneus (which showed a task-related decrease of brain activation) were significantly correlated with intelligence in the total sample (*r*[38] = .41, *p* = .01), suggesting that a stronger decrease of brain activation is related to lower visuo-spatial intelligence. This correlation was similar for males (*r*[18] = .41, *p* = .07) and females (*r*[18] = .42, *p* = .07; see Figure 3).

### Parametric analyses related to task difficulty

**Figure 4:**
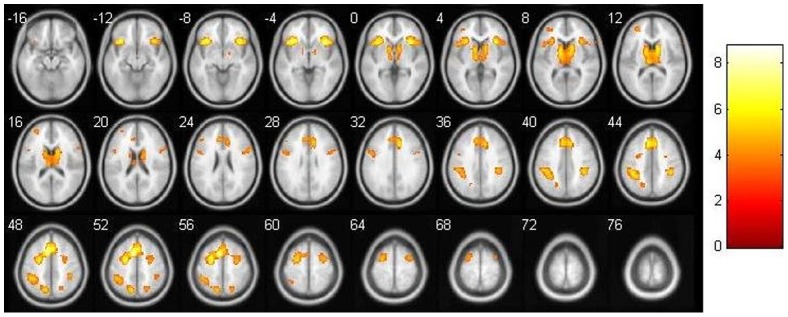
Effects of task difficulty. Areas showing an increase of brain activation with an increase of task difficulty (i.e, rotation angle; *p*<.05, FWE corrected).

We found several brain areas where activation was positively correlated with task difficulty (i.e., rotation angle) including inferior frontal and inferior parietal regions (see Table 2 and Figure 4). These areas did not differ between males and females. There were no areas where activation decreased with increasing rotation angle.

**Table 2:** Task-difficulty related activation. Presented are significant clusters identified with task difficulty (rotation angle) as regressor of interest (*p*<.05, FWE corrected, k>50).

Coordinates			*P*_Cluster_ (FWE)	*T*_Peak_	Cluster size (*k*)	Location
x	Y	z				
−9	11	52	.000	8.76	183	Supplementary motor area (SMA)
36	20	−5	.000	7.41	85	Right inferior frontal
−33	23	−5	.000	7.14	100	Left inferior frontal
−24	−4	55	.000	6.43	56	Left frontal

#### Correlations with intelligence

**Figure 5:**
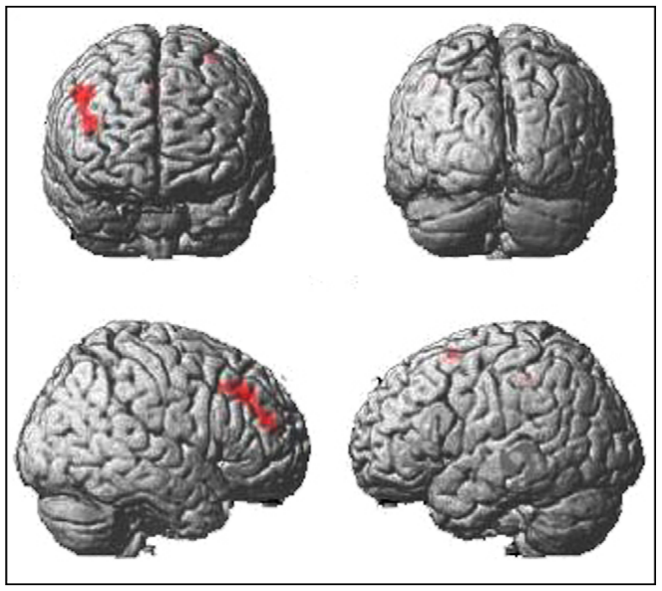
Interaction of task difficulty and intelligence. Areas showing an increase of brain activation with an increase of task difficulty (i.e., rotation angle) for people with higher visuo-spatial intelligence (*p*<10^−3^, uncorrected).

When visuo-spatial intelligence was entered as a covariate into the analysis of task difficulty, three clusters where intelligence influenced the relationship between rotation angle and activation were observed (Table 3). This interaction is due to a stronger increase in activation for participants with higher visuo-spatial intelligence when performing mental rotation of high task-difficulty (see Figure 5). Separate analyses for both genders revealed significant clusters only for females but not for males. They spanned right superior parietal [*p*_Cluster_ = .02 (uncorrected); x, y, z: 36, −73, 49; *T*_peak_ = 4.67, k = 66] and right frontal areas [*p*_Cluster_ = .04 (uncorrected); x, y, z: 57, 26, 31; *T*_peak_ = 4.13, k = 50] as well as the SMA [*p*_Cluster_ = .01 (uncorrected); x, y, z: 3, 20, 61; *T*_peak_ = 4.54, k = 82].

**Table 3:** Intelligence x task difficulty. Significant clusters for the interaction between visuo-spatial intelligence and task difficulty (*p*<.001, uncorrected; p_Cluster_[FWE]<.05, k>50).

Coordinates			*P*_Cluster_ (FWE)	*T*_Peak_	Cluster size (*k*)	Location
x	Y	z				
45	47	22	.002	6.18	241	Right frontal
6	32	46	.002	4.05	253	Right frontal
36	−73	49	.011	4.20	167	Right superior parietal

## Discussion

This study was primarily designed to investigate the neural efficiency hypothesis (NEH) in the visuo-spatial domain by means of fMRI. As visuo-spatial tasks commonly reveal substantial gender differences, the second aim of this study was to examine to which extent the NEH is moderated by gender.

The visuo-spatial task used in the current study was a mental rotation task adapted from Posner's classic letter matching paradigm [10], [11]. We found activation in a widespread and complex neural network, mainly including frontal and parietal areas, which nicely fits into previous research (e.g. [3], [39]). As expected, we also found an effect of rotation angle on behavioral and activation measures, indicating that task difficulty increased with rotation angle.

### The relationship between intelligence and brain activation

Higher visuo-spatial intelligence was related to better performance in the mental rotation task. At the neurophysiological level, intelligence was not related to any increase of brain activation in task-sensitive areas, but rather to a lower decrease of brain activation in an area of the posterior cingulate and the precuneus. This area has been repeatedly related to the default mode network of the brain [45]–[47], a network that is activated when no goal-directed information processing takes place. It has been shown that the more demanding a task, the stronger the default network is deactivated [48], [49]. Therefore, a possible interpretation of our finding would be that less intelligent participants faced greater cognitive demands by the task than more intelligent participants.

Activation within the default mode network has been explained by referring to internally focused thought processes, such as mind-wandering [50], [51] and retrieval from autobiographical memory, future thinking and theory of mind [52]. In the context of cognitive processing, activation in the default mode network has been linked to “task-unrelated thoughts”, internal thought processes that have to be abandoned when resources are needed for cognitive processing [53]. When less intelligent participants needed more resources for solving the tasks they might also have had less task-unrelated thoughts and therefore less activation in their default mode network, as compared to more intelligent participants. The idea that the default mode network might be related to intelligence differences has also been proposed by Van den Heuvel et al. [54] as well as Song et al. [55]. Focusing on connectivity, they found strong negative correlations between intelligence and path lengths in the default mode network, which they interpreted as shorter connections being related to more efficient processing. This finding brings a new aspect to the NEH. Efficient activation might not only be characterized by lower activation in task irrelevant areas, but also by higher activation in resting state areas. Possibly, activation differences in the default-mode network can at least partly account for contradictory findings in the relationship between activation measures and intelligence.

### The role of gender and task difficulty in the relationship between intelligence and brain activation

The second goal of this study was to investigate whether the relationship between intelligence and activation was moderated by the gender of the participants or task difficulty. Males and females in this study did not exhibit any differences with respect to their visuo-spatial intelligence test scores, nor with respect to task performance during fMRI recording. The only general gender difference found with respect to brain activation was a stronger activation of the right lingual gyrus in males, which was also reported for a line orientation task by Clements-Stephens [56]. Previous studies have reported gender differences in brain activation related to the use of different strategies (e.g. [17], [57]). One plausible reason why no general activation differences between females and males were found, might be the absence of gender differences in performance. Also, the employed task was comparatively easy compared to other visuo-spatial tasks (e.g., three-dimensional mental rotation). It might, therefore, be assumed that males and females in this study have used the same strategy to solve the task.

As the rotation angle increases, task difficulty and brain activation increase. The reported interaction between task-difficulty and intelligence suggests that the compensation of increasing task difficulty by an increase of activation might primarily hold true for people with higher visuo-spatial intelligence, and that this was stronger in the female part of the sample. A similar finding has been reported by Preusse et al. [39], who reported stronger activation in an occipital-temporal region with increasing task difficulty in a geometrical analogy task, but only for participants with high fluid intelligence. The authors assume that there might be differences in information processing between people with average and high fluid intelligence. The interaction between difficulty and intelligence in our study was located in right frontal and right inferior parietal regions, areas that have previously been related to mental rotation processes [17], [20], [58]. This suggests that more intelligent people specifically focus their activation on task-related areas when task difficulty increases. It is not clear, however, how less intelligent participants dealt with increasing task demands. One possible explanation might be that they gave up on the task and simply guessed their answers. This, however, appears to be unlikely as the employed spatial task reflected only elementary cognitive demands.

In gender-specific analyses the interaction of task-difficulty and intelligence was only significant for females. The non-significant interaction for males may in part be due to the lower power in the smaller sample. Moreover, more intelligent females may show task-specific activation increases with increasing difficulty to a stronger degree or more consistently than males. Females often perform worse than males in mental rotation tasks, especially when items are presented two-dimensionally and not three-dimensionally (e.g. [59]). Mental rotation tasks with higher task-difficulty hence may have been especially challenging for females. Therefore, more intelligent females might more consistently or more strongly respond with increased task-related brain activation, whereas this mechanism could be less essential for males.

Previous fMRI studies investigating the relationship between brain activation and intelligence reported negative rather than positive correlations between activation and intelligence for females. Jordan and Wüstenberg [57] found stronger activation in some areas for females with low spatial experience as compared to females with high spatial experience. In a study using a working memory task, Tang et al. [29] exclusively reported negative correlations between intelligence measures and brain activation, both for males and for females. This is not in line with our results suggesting adaptation to task difficulty by an increase of task-specific activation. Possibly, a certain task difficulty needs to be met in order to find a positive intelligence-activation relationship, or the negative relationship reported in some studies may reflect the recruitment of less task-relevant areas.

### Further methodological considerations

Most of the existing studies on the neural efficiency hypothesis have been performed with EEG. In one of these EEG studies [11], negative correlations between brain activation (event-related alpha desynchronization; ERD) and intelligence were observed during visuo-spatial tasks in males, a finding that could not be reported in this study, applying fMRI. However, it has to be taken into consideration that the results coming from studies employing from different neurophysiological measurement methods (EEG vs. fMRI) can only be compared with caution. Up to the present, the relationship between event-related ERD in the upper alpha band and the BOLD response is not well understood. Studies performed under resting conditions demonstrated a negative relationship between alpha activity and BOLD response [60], but this might not equally hold true for brain activation during cognitive processes. Increases of alpha activity might not necessarily be indicative of deactivation, as it has recently been related to active inhibition processes [61]–[63] via the blocking of task-irrelevant information processing pathways. Whether there is a positive or negative relationship between alpha power and BOLD signal also seems to depend on the brain area investigated [60], [64], [65]. In a study designed to compare resting state activity in fMRI and EEG, Jann et al. [66] found positive correlations between power in the upper alpha band and the BOLD response in regions associated with the default mode network. It is possible that alpha desynchronization in the EEG during cognitive tasks can be related to a deactivation of the default mode network in fMRI.

### Limitations and prospect

This study used a comparatively simple cognitive task to avoid that less intelligent participants give up working on the task, and to obtain short and well-defined events of cognitive activation. It is possible that more complex items that are more similar to those typically used in measures for the assessment of intelligence might be more adequate when investigating neural efficiency with fMRI. Even in the simple task used here, the typical relationship between intelligence and solution rate as well as reaction time was observed. This, however, poses some limitations to the investigation of neural efficiency by means of fMRI, as longer reaction times can lead to a higher BOLD response [67]. Therefore, an adequate control for subjective task difficulty and reaction times is essential for future studies.

Another challenging factor in neural efficiency research is that there are two main sources from which a relationship between intelligence and brain activation might derive: one is the number of different cortical networks recruited in order to solve cognitive tasks, which is related to the underlying strategies and executive control processes needed to perform a task successfully. Second, neuroanatomical and physiological properties of the brain (e.g. grey matter density) might contribute to the amount of energy that is required for information processing. Possibly, both factors are involved, and further research is needed to assess the contribution of each source separately.

## Conclusions

The present study revealed no clear evidence for a negative relationship between psychometrically determined intelligence and brain activation. However, our results suggest that intelligence might be related to a deactivation of regions involved in the default mode network, which may be indicative of higher task demands for less intelligent people. This higher demand could be associated with the additional recruitment of brain areas that do not have to be consistent across different individuals. As task difficulty increases, brain activation in task-relevant areas was increased in more intelligent individuals, especially in females. They possibly invest more mental effort when tasks get more difficult. These findings may contribute to a more differentiated formulation of the neural efficiency hypothesis, and might also help to better understand gender differences in the visuo-spatial domain.
